# Machine Learning-Based Prediction of Decompensation in Hepatitis B Virus-Related Cirrhosis

**DOI:** 10.3390/diagnostics15212790

**Published:** 2025-11-04

**Authors:** Hsueh-Chun Lin, Meng-Lun Hsieh, Meng-Yu Liu, Chin-Chi Kuo, Shwn-Huey Shieh, Ming-Shun Hsieh, Vivian Chia-Rong Hsieh

**Affiliations:** 1 Department of Health Services Administration, China Medical University, Taichung 406, Taiwan; snowlin@mail.cmu.edu.tw (H.-C.L.); iris523102@gmail.com (M.-Y.L.); shshieh@mail.cmu.edu.tw (S.-H.S.); 2 Division of Gastroenterology, Department of Internal Medicine, Taichung Veterans General Hospital, Taichung 407, Taiwan; morganpolo@gmail.com; 3 Big Data Center, China Medical University Hospital, Taichung 404, Taiwan; fenderkuo@gmail.com; 4 Renal Division, Department of Internal Medicine, China Medical University Hospital, Taichung 404, Taiwan; 5 Department of Nursing, China Medical University Hospital, Taichung 404, Taiwan; 6 Department of Emergency Medicine, Taichung Veterans General Hospital, Taichung 407, Taiwan; edmingshun@gmail.com; 7 School of Medicine, National Yang Ming Chiao Tung University, Hsinchu 300, Taiwan

**Keywords:** HBV-related cirrhosis, decompensation, machine learning, electronic health records, ascites, variceal bleeding, jaundice

## Abstract

**Background/Objectives**: Fatality of cirrhotic patients greatly increases when they progress to the decompensated state. Only a few studies to date have applied machine learning (ML) methods to predict decompensation in cirrhosis patients. In the present study, we attempted to apply self-developed ML models for validating their capability of predicting different complications in hepatitis B virus (HBV)-related cirrhosis patients. **Methods**: Data were extracted from electronic health records of 50,047 patients who were tested and diagnosed with HBV in a tertiary hospital. Four different algorithms (Support Vector Machine (SVM), Logistic Regression (LR), Decision Tree (DT), Random Forest (RF)) were utilized, and a total of 32 ML models were trained and tested to predict variceal bleeding, ascites, jaundice, and multiple complications (≥2 complications) in HBV-related cirrhosis patients. The use of two antiviral drugs were considered: entecavir (ETV) and lamivudine (LAM). Performance of the models was assessed using area under receiver operating characteristic curve (AUROC) and accuracy score. **Results**: SVM and RF classifications produced the best overall predictions for decompensation in HBV-related cirrhosis patients, with AUROCs ranging from 0.85 to 0.93 and accuracy scores between 0.77 to 0.88 for ascites, jaundice, and multiple complications. The SVM and LR algorithms generated the best performance in differentiating ascites among ETV users, with AUROC of 0.93 and 0.92 and accuracy of 0.88 and 0.86, respectively. Antiviral treatment (type, length of use, adherence), and other routinely collected clinical information may serve as informative markers in differentiating decompensated cirrhosis. **Conclusions**: ML-based prediction of decompensation using electronic health records may assist clinicians in decision making. Findings of this study also underline the impact of antiviral therapy as a key predictor for decompensation.

## 1. Introduction

Chronic hepatitis B (CHB) infection remains one of the most prevalent communicable diseases worldwide, especially in Asia. In Taiwan, approximately 13.7% of the population are CHB-infected [1]. A significant number of individuals with CHB infection eventually advance to cirrhosis, and in severe cases, this condition can further deteriorate into life-threatening decompensation, characterized by severe complications [2]. These complications include jaundice, ascites, variceal bleeding, hepatorenal syndrome (HRS), hepatic encephalopathy (HE), and spontaneous bacterial peritonitis (SBP). Cirrhosis also significantly increases the risk of developing hepatocellular carcinoma (HCC), with most HCC patients having a history of cirrhosis [3]. Therefore, each of these conditions is a critical consequence for cirrhosis patients and requires specific treatments in disease management; however, by promptly detecting and addressing these conditions, there is a potential to significantly enhance the survival rates of these patients and alleviate their burden of disease [4].

In recent years, there has been a remarkable development in the field of big data and machine learning (ML) algorithms, particularly in the area of disease prediction and identification of valuable biomarkers. This progress has had a significant impact on the study of liver diseases, as evidenced by numerous research endeavors. One notable Canadian study utilized ML algorithms to analyze multi-center patient data, resulting in the successful detection of all-cause advanced hepatic fibrosis with high classification and prediction capabilities [5].

There have also been multiple studies employing ML techniques to predict fatty liver disease, yielding promising results. In a particular study involving 513 subjects, body composition and anthropometric data were utilized, and among the different ML methods employed, the random forest (RF) algorithm demonstrated the highest accuracy, achieving an impressive 82% accuracy rate [6]. Similarly, in a Taiwanese study using clinical and laboratory data from 31,930 individuals, a model using the extreme gradient boosting algorithm successfully diagnosed fatty liver disease with an accuracy of 0.833 and achieved an area under the receiver operating characteristic curve (AUROC) of 0.882 [7]. Body mass index was found to be the most influential feature in this study.

In terms of HCC recurrence, ML has been used by a number of studies to provide predictive models and inform preventative measures helpful for recurrence management [8,9,10,11]. Additionally, Farghaly et al. and Edeh et al. have integrated ML approaches such as Naïve Bayes and ensemble models in studying hepatitis C virus (HCV) infection [12,13].

Decompensation remains a significant mortality risk factor in cirrhosis patients, with many even experiencing multiple complications, indicating that they can have more than one of the conditions mentioned above [14]. However, only a few studies to date have applied ML methods on this group of patients. Using a RF model, the prediction of 1-year survival in cirrhosis patients after transjugular intrahepatic portosystemic shunt was better than that achieved by existing prognostic scores [15]. Similarly, ML models have been used to predict all-cause mortality in cirrhosis patients, with clinical variables and laboratory test results as key features [16,17,18]. Other studies have focused on specific endpoints for the prediction of decompensation among cirrhosis patients, such as HRS [19] and esophageal varices [20,21,22]. More recent studies have leveraged the use of ML techniques to predict overall decompensation, defined as the presence of ascites, HE, jaundice, variceal bleeding, or SBP, with promising performance [23,24]. A table is provided to summarize the above-mentioned studies (Table 1).

In this study, we aimed to apply self-developed ML models for differentiating four different decompensation states (variceal bleeding, ascites, jaundice, and multiple complications) in patients with HBV-related cirrhosis and for identifying informative features that can enable timely intervention for these patients. To better emulate real-life scenarios, we considered both individual complications and multiple complications as our prediction endpoints. This article is structured into five main sections: Introduction, Materials and Methods, Results, Discussion, and Conclusions.

Our goal is to provide clinicians with a tool that offers more accurate and timely prediction of decompensation, which will better inform prognosis and guide management.

## 2. Materials and Methods

We developed and validated self-developed ML models to predict various complications in a large cohort of 50,047 hepatitis B virus (HBV)-related cirrhosis patients in a tertiary hospital setting.

### 2.1. Data Source

This study utilized de-identified electronic health records from hepatitis B patients with cirrhosis between 1 January 2003 and 31 December 2017 which included information on the patients’ disease history (International Classification of Diseases, Ninth and Tenth Editions, Clinical Modification; ICD-9-CM and ICD-10-CM codes), prescription record (name of medication, dose, date, and duration), laboratory test (name of test, date performed, value), and demographics (age, sex, weight, and height). The data were obtained from a 2202-bed medical center in central Taiwan and accessed in a regulated setting between 22 October 2018 and 31 May 2021 for research purposes.

Extracted laboratory test items included HBV infection markers (HBV DNA, HBsAg, anti-HBs, HBeAg, anti-HBe) for identification of the cirrhosis patients, liver biochemical tests (alanine transaminase (ALT), aspartate transaminase (AST), total bilirubin, albumin, prothrombin time, platelet count), and other related laboratory tests (creatinine, sodium, fasting glucose, alpha-fetoprotein (AFP)). Five different antiviral drugs were initially identified: lamivudine (LAM), adefovir, entecavir (ETV), telbivudine, and tenofovir (TDF). Aside from the type of antiviral drug used, the length of antiviral treatment and patient’s medication adherence were also considered. At a result, a total of 14 features including patient demographics (age and sex), liver biochemical tests, other related laboratory tests, and medication adherence (measured as length of treatment (days) and medication possession ratio) were inputted for their potential impact on the occurrence of decompensation in cirrhosis patients (Table S1). Medication possession ratio (MPR) is calculated as a ratio of the number of days the patient has supply of medication over the total number of observed days. An MPR of greater than 0.8 is indicative of highly adherent behavior and an MPR of 0 indicates no use.

All data were analyzed anonymously. This study was performed according to the Declaration of Helsinki and was approved by the institutional review board of China Medical University Hospital (CMUH107-REC2-105). Informed consent to participate was waived by the same institutional review board since only de-identified records were used, and patient confidentiality was protected by anonymizing all data before use.

### 2.2. Study Population

Between 2003 and 2017, a total of 118,424 HBV antigen reactive reports were identified. These reports belonged to 50,047 patients who were tested and diagnosed with HBV (HBV antigen reactive) from both outpatient and inpatient encounters in the medical center. Only patients aged 20 or over at the time of the report were included (*n* = 49,418). We subsequently verified their incident decompensation after cirrhosis diagnosis (diagnostic codes as seen in Table S2), and their laboratory data availability. To ensure the prediction ability of the models, we focused on individuals who consistently used a single antiviral drug rather than those with frequent changes in their treatment regimen (*n* = 135). We are convinced that, in normal circumstances, there should not be frequent changes to the antiviral therapy under the national health insurance scheme due to concerns of drug resistance. As TDF became eligible for reimbursement in June 2011 for HBV treatment, the number of patients using this drug continuously throughout our data collection period (2003–2017) was limited, leading to their exclusion from this study. However, ETV and LAM had been eligible for reimbursement since 2006 and 1999, respectively, resulting in a larger sample with persistent use. Patients who were prescribed with adefovir and telbivudine were also minimal.

**Figure 1:**
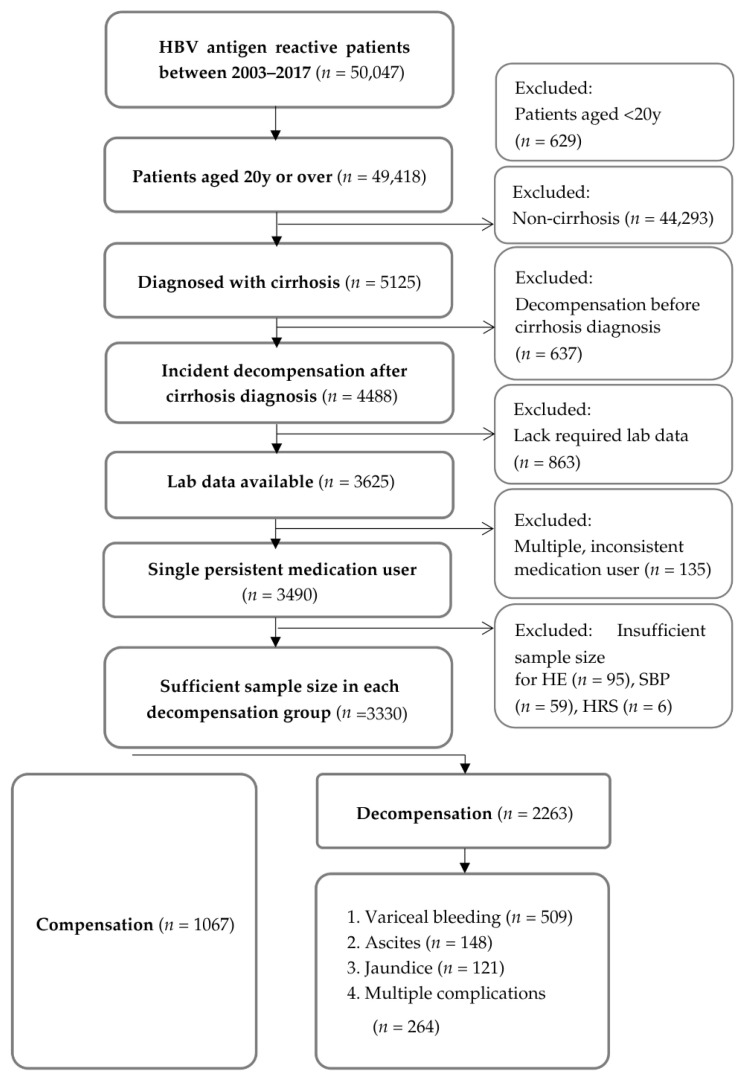
Selection flowchart for patients identified from the electronic health records. Abbreviations: HBV—hepatitis B virus; HE—hepatic encephalopathy; SBP—spontaneous bacterial peritonitis; HRS—hepatorenal syndrome; HCC—hepatocellular carcinoma.

Prediction endpoints considered were variceal bleeding, ascites, jaundice, and multiple complications as defined with the patients’ diagnostic codes (Table S2). For the purpose of this study, multiple complications would indicate at least 2 of the complications occurring concurrently. We ensured an adequate sample size (*n* ≥ 100) for each of the four decompensation groups (ascites, variceal bleeding, jaundice, and multiple complications) before conducting further analysis. HE, SBP, and HRS all had sample sizes of less than 100 patients and were thus excluded. Figure 1 illustrates the steps involved in our subject selection process.

### 2.3. Data Preprocessing

Prior to utilizing the data obtained from the electronic health records, outliers were first identified by applying upper (Q3 + 1.5*IQR) and lower limits (Q1 − 1.5*IQR), and these outliers were treated as equivalent to missing values [25]. To handle the missing values, we used Python 3.6’s SciPy package, version 1.7.0 and imputed them based on the gamma distribution of the parameters, considering the inherent nature of their original distribution, which was non-normal. This approach allowed us to impute values that preserved the original distribution, as opposed to using mean, median, or mode values, which may lead to data points clustering around a central value and exhibit central tendency. By preserving the data’s dispersion and maintaining its variability, this method can potentially enhance model performance, especially in heterogeneous clinical datasets [26]. We iteratively repeated this data preprocessing process, creating various combinations of numerical data for training, and selected the model with the best performance for prediction.

Given the considerable variation in measurement scales among the parameters or features, we implemented feature scaling methods to achieve normalization. Specifically, we employed min-max scaling, which transformed the values of different features into a standardized range between 0 and 1 based on their respective minimum and maximum values. To establish the training dataset, we utilized 80% of the data, which was arbitrarily extracted from the electronic health records. This split ratio was used with consideration of the limitations of our dataset, which was not large and imbalanced [27,28]. The specific methods used for data normalization and splitting are detailed in our earlier publication, which provides a comprehensive guide to building the clinical decision support system [29].

### 2.4. Feature Selection and Balancing Datasets

Using the preprocessed data, we performed feature selection to eliminate redundant or irrelevant features and optimize the model’s performance. We employed univariate feature selection via the filter method, evaluating each feature individually. Specifically, we applied the Chi-square test and Student’s *t*-test to determine statistically significant differences between categorical and continuous variables related to the complications, respectively [29,30]. Features that were statistically significant (*p*-value < 0.05) were used to create different groups of features to enhance the model’s performance in cross-validation.

Next, one-to-one matching between compensated and decompensated patients was conducted for each prediction endpoint. This matching process was crucial to ensure balanced datasets for training, preventing predictability from favoring the group with a larger sample size or higher probability. Additionally, we took measures to verify an equal proportion of antiviral users between the two patient groups.

### 2.5. Machine Learning Models

The “scikit-learn” 1.5.1 package in Python version 3.6 was utilized to implement the four ML algorithms used in this study: Support Vector Machine (SVM), Logistic Regression (LR), Decision Tree (DT), and RF. We selected these algorithms for this study due to their widespread use and their potential effectiveness in classifying the given data. SVM is a supervised algorithm that excels at determining optimal decision boundaries between different classes. Its primary goal is to maximize the separation, or margins, between classes by identifying the support vectors that are closest to the decision boundary. SVM is versatile in handling both linear and non-linear classifications and is well-suited for high-dimensional data. In this study, the classifier utilized the radial basis function kernel to define support vectors for separating feature dimensions.

LR, a statistical modeling technique, establishes the relationship between a dependent variable (binary outcome) and independent variables (input features) using the logistic function. During training, the model adjusts its parameters to maximize the likelihood of the observed data given the predicted probabilities. Logistic regression is widely used due to its simplicity, interpretability, and effectiveness in various applications.

Another widely employed ML algorithm is DT, which partitions data into subsets based on decision rules it generates. Decision rules, or branches, are created at nodes depending on the input feature values using specific criteria. This process continues recursively until an optimal ‘tree’ is formed, with well-defined splits and decision rules that effectively segregate the classes of data.

RF combines multiple decision trees by using the bagging technique. Each decision tree is trained independently and makes predictions based on the feature values. The final prediction is determined by aggregating the predictions from all individual decision trees, either through voting (for classification) or averaging (for regression). RF generally outperforms single decision trees, exhibiting higher accuracy and a reduced risk of overfitting. Hyperparameters of the algorithms used in this study are presented in Table 2.

Our aim was to select the best performing model in predicting decompensation among HBV-related cirrhosis patients. For this reason, these four ML algorithms were chosen in this study for their distinct classification approaches. For more in-depth information on the algorithms used, please refer to our previously published research [29].

### 2.6. Training

Ten-fold cross-validation was used in the training process to evaluate the model’s accuracy. Given our limited data, we initially trained the models using all features and explored various combinations without applying feature selection. This trial-and-error approach allowed us to experiment with different feature sets. However, the results were not consistently strong across all models, which led us to ultimately pursue feature selection as a more effective strategy. For transparency, we have included these preliminary results in the Supplementary Materials for reference (Table S3). After each fold, an accuracy score was calculated. The models with the highest average accuracy after 10-fold cross-validation were advanced for validation.

### 2.7. Validation

Performance evaluation metrics used to compare the validated ML models included AUROC and accuracy. Accuracy measures the proportion of true positive and true negative predictions, while AUROC plots sensitivity against 1-specificity, making it a key evaluation metric for ML models. Higher value of all these metrics would imply better model performance. An AUROC value between 0.7 and 0.8 is deemed acceptable based on recent related studies and clinical applications [23] and any level above would indicate a good (0.8–0.9) or even exceptional performance (0.9–1.0).

## 3. Results

We tested a total of 32 ML models, with 4 models dedicated to each of the 4 decompensation endpoints and each endpoint was examined for 2 separate groups of antiviral users (LAM and ETV). After balancing datasets, we have obtained patient populations used to train and test the algorithms specific to each prediction endpoint and antiviral therapy. Table 3 lists the sizes of patient samples used for each prediction outcome in the training and validation sets (a completely separate, unused 20% of data to test the model’s ability to generalize to new data). Tables S4–S7 provide the characteristic profiles of patients included in the classification models.

### 3.1. Variceal Bleeding

For LAM users, data from 652 patients (326 positive, 326 negative) were used to train and 164 to validate (82 positive, 82 negative). Mean age of patients was 52 years and male predominance was observed (76%). Eight features selected include AST, total bilirubin, albumin, fasting glucose, creatinine, prothrombin time, platelet, and medication possession ratio (Table S8). Highest AUROC and accuracy were achieved in the SVM model, with scores of 0.71 and 0.70, respectively (Table 4). The LR model also performed well with an AUROC of 0.71 and accuracy of 0.68.

For ETV users, data from 748 patients (374 positive, 374 negative) were used to train and 186 to validate. Demographics profile was similar to that of LAM users (i.e., male predominance and mean age of 52 years). Eleven features selected include sex, AST, total bilirubin, albumin, fasting sugar, creatinine, prothrombin time, platelet, AFP, length of treatment, and medication possession ratio. After testing the trained models, we observed that the four different classifiers showed an acceptable overall performance, with AUROC ranging from 0.63 to 0.79. Similar to LAM users, SVM model exhibited the best prediction ability (AUROC: 0.79, accuracy: 0.72).

### 3.2. Ascites

Mean age of patients with ascites was between 55 and 56 years, while majority (64 to 65%) of them were males. For LAM users, data from 178 patients were used to train and 44 to validate. Ten features selected are shown in Table S8. RF model had the best AUROC of 0.76 (accuracy: 0.70) among the four models. For ETV users, data from 222 patients were used to train and 56 to validate. Ten features were also selected. Unlike LAM users, LR showed the best performance (AUROC: 0.93, accuracy: 0.88), closely followed by the SVM model (AUROC: 0.92, accuracy: 0.86).

### 3.3. Jaundice

Eighty percent of jaundice patients under the LAM regimen were male, with a mean age of 53 years. Data from 120 patients were used to train and 30 to validate. The RF model achieved an exceptional performance (AUROC: 0.91, accuracy: 0.87), followed by the SVM model (AUROC: 0.87, accuracy: 0.77).

The 9 features selected for LAM and ETV models were identical: ALT, AST, total bilirubin, albumin, fasting glucose, prothrombin time, sodium, length of treatment, and medication possession ratio (Table S8). Under the ETV treatment, 79% of jaundice patients were male and their mean age was 52 years. Data from 172 patients were used to train and 44 to validate. Among the 4 algorithms, the RF model exhibited the highest AUROC and accuracy scores of 0.81 and 0.73, respectively.

### 3.4. Multiple Complications

For cirrhosis patients who developed multiple complications, SVM was able to predict the endpoint with an AUROC of 0.85 and accuracy of 0.77 under the ETV regimen using 13 selected features (Table S8). For patients under the LAM therapy, our best model (LR) was only able to achieve an AUROC of 0.74 and accuracy score of 0.68 with 12 features. SVM model also showed an AUROC of 0.73 and accuracy of 0.71. Demographics of patients with multiple complications were similar to that of the other complication groups.

Overall performance and the ranking of the models is summarized in Table 5. Under LAM therapy, RF algorithms performed the best in predicting jaundice and ascites. Under ETV regimen, LR algorithm performed the best in predicting ascites, while SVM algorithm was best in predicting variceal bleeding and multiple complications.

## 4. Discussion

This was one of the few current studies employing ML methods to predict decompensation, as well as multiple complications in HBV-related cirrhosis patients. Using 15-year electronic health record data, we tested four different ML approaches and demonstrated that SVM and RF classifications produced the best overall predictions for decompensation in HBV-related cirrhosis patients, with AUROC of 0.85 to 0.93 and accuracy of 0.77 to 0.88 for ascites, jaundice, and multiple complications. This result closely resembles the performance that was previously achieved in ML models from past studies examining similar endpoints. Using a RF classification, Dong et al. effectively identified at-risk cirrhosis patient for esophageal varices, with an AUROC of 0.84 and 0.82 in the training and validation sets, respectively [22]. In another recent study on predicting prior decompensation, a RF model was able to achieve AUROCs of 0.95 and 0.87 on training and test data, respectively [23]. Other studies demonstrated comparable performance in predicting non-specific liver disease and liver disease-related mortality using ML and statistical methods, with reported accuracies of over 0.8 and the best AUROCs ranging from 0.8 to 0.9 [31,32,33].

Conversely, some existing studies have been able to predict specific outcomes with high accuracy in patients with liver cirrhosis using neural network models. Using an Artificial Neural Network model, with key input data from clinical and biochemical parameters, the model achieved a high AUC of 0.959 in predicting variceal bleeding [20]. Another study predicted broader, longer-term outcomes of decompensation and liver-related death using a Convolutional Neural Network model integrating electrocardiogram data, resulting in a high AUC of 0.933 [24]. The adoption of deep learning models in these studies likely contributed to the high model performance by better handling non-linear relationships within the data and finding the “hidden” signals in the complex clinical data.

Extreme Gradient Boosting (XGBoost) algorithms have also been used in predicting decompensation with comparable results. A study predicting HRS showed high predictive performance, with AUCs of 0.832 in the training set and 0.8415 in the validation set [19]. Another study predicting variceal bleeding using the same algorithm resulted in high accuracies of 93.7% in the internal validation set and 85.7% in the external validation set [21]. These consistently high-performance metrics across different outcomes highlight XGBoost as a potentially robust and generalizable tool for risk stratification in advanced liver disease.

In comparison, the SVM and RF models in our study achieved AUROCs of 0.85 and 0.91, respectively, and accuracy scores of 0.77 and 0.87, which may adequately meet physicians’ expectations for clinical decision support. Generally, SVM and RF outperform LR and DT, potentially due to their unique classification properties and advantages. RF generally outperforms DT because it is an ensemble learning technique that aggregates a large number of decision trees, which reduces variance compared to single decision trees on the same dataset [34,35,36]. When comparing the performance of LR and RF, RF has also been shown to demonstrate better accuracy, especially as the number of features and dimensionality increase [37]. SVM is frequently utilized in disease prediction, with SVM and RF often demonstrating the highest accuracies [34,38].

Among all tested ML models, however, the LR algorithm differentiating ascites in ETV users had the best performance (AUROC: 0.93 (95% CI: 0.86–0.99), accuracy: 0.88, which is closely followed by the SVM model that achieved an AUROC of 0.92 and accuracy of 0.86. We confirmed that the commonly tested clinical and demographic markers in cirrhosis patients, including ALT, total bilirubin, albumin, platelet count, and age, are strong risk factors for decompensation. This is similar to the findings of previous studies where they identified that ALT, platelet count, total bilirubin, albumin, prothrombin time were among the significant independent predictors of hepatic decompensation [20,39]. Several ML studies have also identified serum albumin, ALT, platelet count, and hemoglobin as significant risk factors for decompensated cirrhosis [16,20,22]. With this result, we are optimistic that ML algorithms may be valuable tools for facilitating timely prevention and management of ascites secondary to HBV-related cirrhosis.

Our second-best performing model was the SVM model in predicting multiple complications in patients under the ETV regimen. We hypothesize that the need for more features to predict multiple complications could be explained in part by the noise in the patient characteristics. The prediction results may be influenced by various underlying factors associated with the presence of multiple complications, which could potentially impact accuracy. Nevertheless, this is one of the first studies to consider multiple complications as the outcome of interest. It is important to note that future studies will be required to validate the outcomes of our model.

### 4.1. Theoretical and Practical Implications

Given our study results, ML-based prediction models may serve as a valuable aid in the clinical decision-making process for predicting decompensation. After examining our ML models, we found that AST, total bilirubin, albumin, and prothrombin time were the most frequently selected features, appearing in all complications for both medication groups. Unsurprisingly, these four biomarkers are common liver biochemical tests that indicate the level of liver tissue damage and severity. This study thus validates the clinical relevance of established liver damage markers by demonstrating their consistent importance across various complications, regardless of the specific antiviral used. Routine monitoring of these markers is thus highly recommended when assessing the risk of decompensation in practice.

Medication also emerged as an important predictor of almost all decompensation outcomes we examined. Not only the specific type of antiviral drug, but also the length of treatment and the medication possession ratio of the prescribed regimen were identified as critical factors in the occurrence of decompensation. This suggests that antiviral management itself—including adherence and regimen choice—is an independent and significant determinant of the clinical course in HBV-related cirrhosis patients. ETV has shown superior virologic and biochemical efficacy compared to LAM, while long-term use of LAM is associated with a higher risk of developing viral resistance, reducing its ability to suppress virus replication [40,41]. Our results also highlight that both the proper length of antiviral treatment prescribed by clinicians and patient adherence to those orders are significant factors in preventing decompensation. Current available evidence strongly supports the notion that maintaining sustained adherence to antiviral treatment in patients with HBV and HBV-related cirrhosis plays a protective role in mitigating the risk of decompensation and mortality [42,43]. It emphasizes the need for aggressive patient adherence programs and minimizing treatment interruptions. We thus believe this is one of the strengths of our work as this information was not considered in previous studies with ML approaches, which has been extensively examined in epidemiological studies.

### 4.2. Limitations of Study

Nevertheless, this study is not without caveats. First of all, our data were limited to one tertiary hospital which made our patient sample relatively homogeneous. For certain complications such as HE and SBP, the available sample sizes were insufficient to adequately train and validate the models. Secondly, we did not consider cirrhosis outside of HBV etiology as it was our primary intention to focus on this specific group of patients. This decision was made deliberately to maintain the context and scope of our research. Furthermore, we were unable to extract an adequate patient sample for individuals under the TDF regimen to include in this investigation, since it was available much later than ETV and LAM. As a result, the applicability of the features and developed models for TDF usage, as well as for individuals using multiple (interchanging) antiviral drugs, requires further examination in future studies. Our study results can serve as a guiding example for future classification models, highlighting antiviral treatment and medication adherence as probable predictors of decompensation. Additionally, we did not validate our different models on a completely independent new dataset from an external source. This is due to data privacy and confidentiality concerns, which prevented the exchange and access of information across different healthcare providers. Nevertheless, many previous studies using ML algorithms to predict disease outcomes have followed a similar approach of dividing a dataset into two parts, one for training and one for testing [44,45]. Finally, there are limitations of univariate feature selection, including its inability to account for interactions between features and oversimplifying the ranking process by evaluating features individually. This approach might overlook potential combined effects, leading to reduced model performance due to the exclusion of significant interactions [30,46].

## 5. Conclusions

In conclusion, we have validated self-developed ML models from electronic health records that may well predict the risk of decompensation among HBV-related cirrhosis patients. Routinely collected clinical information and treatment regimen may serve as informative features in differentiating potential complications in this patient population. Findings of this study also underline the prospect for adopting machine-learning based methods in assessing the risk of liver-related outcomes.

## Figures and Tables

**Table 1:** Analysis of the studies employing ML techniques to predict liver-related outcomes in cirrhosis patients.

Prediction Endpoint	Study	Subjects	Method	Results
One-year survival	Da et al. (2024) [15]	Cirrhosis patients after transjugular intrahepatic portosystemic shunt	Random Forest	The Random Forest model better predicted 1-year survival compared to existing prognostic scores (e.g., MELD), with an AUC of 0.82 (0.72–0.91).
All-cause mortality	Guo et al. (2021) [16]	Cirrhosis patients	Deep Neural Networks (DNN), Random Forest, Logistic Regression	The DNN model achieved an AUC of 0.88, 0.86, and 0.85 for 90, 180, and 365-day mortality, respectively.
	Kanwal et al. (2020) [17]	Cirrhosis patients	Gradient Descent Boosting, Logistic Regression with Least Absolute Shrinkage and Selection Operator (LASSO) regularization, Partial Path Logistic Model	The Gradient Descent Boosting model achieved an AUC of 0.81 (0.80–0.82), outperforming logistic regression with LASSO regularization (AUC: 0.78) and the Partial Path Logistic model (AUC: 0.78).
	Guo et al. (2025) [18]	Participants with 1 or more of the chronic liver disease risk factors	Gradient-Boosting	The ML model predicted a 10-year risk of cirrhosis-related admission, death, or HCC presentation with AUCs of 0.84 and 0.861 in the training and validation cohorts, respectively.
HRS	Yao et al. (2025) [19]	Cirrhosis patients	LASSO regression, Extreme-Gradient Boosting, Random Forest	The ML model showed high predictive performance for the development of HRS, with AUCs of 0.832 in the training set and 0.8415 in the validation set.
Esophageal varices/variceal bleeding	Hou et al. (2023) [20]	Cirrhosis patients.	Artificial Neural Network	The model was able to accurately predict the risk of variceal bleeding with an AUC of 0.959.
	Agarwal et al. (2021) [21]	Patients with compensated advanced chronic liver disease	Extreme-Gradient Boosting	The ML model predicted future variceal bleeding, achieving an accuracy of 98.7%, 93.7%, 85.7% in the derivation, internal validation, and external validation set.
	Dong et al. (2019) [22]	Cirrhosis patients	Random Forest	The derived ML-based scoring system achieved AUCs of 0.84 and 0.82 in the training and validation sets, superior to existing non-invasive indices.
Decompensation (ascites, HE, jaundice, variceal bleeding, or SBP)	Müller et al. (2025) [23]	Cirrhosis patients.	Decision Tree, Random Forest, Support Vector Machines, Neural Networks	Random Forest model achieved an AUC of 0.87 and an accuracy of 70.5% on test data for retrospective prediction.
	Ahn et al. (2025) [24]	Cirrhosis patients	AI-Cirrhosis-ECG (ACE) Score (Convolutional Neural Network model using ECG features).	The ACE score accurately identifies hepatic decompensation with an AUC of 0.933.

**Table 2:** Algorithm hyperparameters used in this study.

Algorithm	Hyperparameters	Definition	Value Range
SVM	C	Regularization parameter	1.0
	kernel	Radial basis function kernel algorithm	RBF
	degree	Degree of the polynomial kernel function	3
	gamma	Kernel coefficient for ‘RBF’	SCALE
	tol	Tolerance for stopping criterion	0.001
	cache_size	Size of the kernel cache	200
	decision_function_shape	One-versus-rest function	OVR
LR	penalty	Specify the norm of the penalty	L2
	tol	Tolerance for stopping criteria	0.0001
	C	Inverse of regularization strength	1.0
	solver	Algorithm to use in the optimization problem	LBFGS
	max_iter	Number of iterations	1000
	multi_class	A binary problem is fit for each label	OVR
DT	criterion	Quality of a split for supporting the impurity criteria	GINI
	splitter	Strategy used to choose the split at each node	BEST
	min_samples_split	The minimum number of samples required to split an internal node	2
	min_samples_leaf	The minimum number of samples required to be at a leaf node	1
RF	n_estimators	Number of trees	100
	criterion	Quality of a split for supporting the impurity criteria	GINI
	min_samples_split	The minimum number of samples required to split an internal node	2
	min_samples_leaf	The minimum number of samples required to be at a leaf node	1
	max_features	The number of features to consider when looking for the best split (square root of number of features)	SQRT
	bootstrap	Using bootstrap samples when building trees	TRUE

**Table 3:** Sample sizes used in the training and validation sets.

	Decompensation					Compensation		Total	
	User (1)	Nonuser (2)	Subtotal (1) + (2) = (3)	80% for Training (4)	20% for Validation (5)	Matched ^1^ Training Set (6)	Matched ^1^ Validation Set (7)	Training (4) + (6)	Validation (5) + (7)
Variceal bleeding									
LAM	42	366	408	326	82	326	82	652	164
ETV	101	366	467	374	93	374	93	748	186
Ascites									
LAM	9	102	111	89	22	89	22	178	44
ETV	37	102	139	111	28	111	28	222	56
Jaundice									
LAM	13	62	75	60	15	60	15	120	30
ETV	46	62	108	86	22	86	22	172	44
Multiple complications									
LAM	11	190	201	161	40	161	40	322	80
ETV	63	190	253	202	51	202	51	404	102

^1^ Decompensated group and compensated group were matched at 1:1 ratio (i.e., (4):(6) and (5):(7)).

**Table 4:** Comparison of model performance for different medication groups after validation.

	Lamivudine (LAM)	Entecavir (ETV)
Variceal bleeding	*n* = 326, *n* = 326 (8 features selected)	*n* = 374, *n* = 374 (11 features selected)
	AUROC: SVM/LR (0.71) > RF > DT Accuracy: SVM (0.70) > LR > RF > DT	AUROC: SVM/RF (0.79) > LR > DT Accuracy: SVM (0.72) > RF > LR/DT
Ascites	*n* = 89, *n* = 89 (10 features selected)	*n* = 111, *n* = 111 (10 features selected)
	AUROC: RF (0.76) > SVM > LR > DT Accuracy: SVM (0.73) > LR/RF > DT	AUROC: LR (0.93) > SVM/RF > DT Accuracy: LR (0.88) > SVM > RF > DT
Jaundice	*n* = 60, *n* = 60 (9 features selected)	*n* = 86, *n* = 86 (9 features selected)
	AUC: RF (0.91) > SVM > LR > DT Accuracy: RF (0.87) > SVM > DT > LR	AUROC: RF (0.81) > SVM > LR > DT Accuracy: RF (0.73) > SVM > DT > LR
Multiple complications	*n* = 161, *n* = 161 (12 features selected)	*n* = 202, *n* = 202 (13 features selected)
	AUROC: LR (0.74) > SVM > RF > DT Accuracy: RF/SVM (0.71) > LR > DT	AUROC: SVM (0.85) > LR > RF > DT Accuracy: SVM (0.77) > RF > LR > DT

Abbreviations: AUROC = area under receiver operating characteristic curve; SVM = support vector machine; LR = logistic regression; RF = random forest; DT = decision tree.

**Table 5:** Ranking of model output for each decompensation endpoint by antiviral drug.

	Variceal Bleeding	Ascites	Jaundice	Multiple Complications
Lamivudine (LAM)				
Model	SVM	RF	RF	LR
Features	8	10	9	12
AUROC (95% CI)	0.71 (0.63–0.79)	0.76 (0.62–0.91)	0.91 (0.80–0.99)	0.74 (0.63–0.85)
Accuracy	0.70	0.70	0.87	0.68
Ranking	3	2	1	4
Entecavir (ETV)				
Model	SVM	LR	RF	SVM
Features	11	10	9	13
AUROC (95% CI)	0.79 (0.72–0.85)	0.93 (0.86–0.99)	0.81 (0.68–0.94)	0.85 (0.77–0.93)
Accuracy	0.72	0.88	0.73	0.77
Ranking	4	1	3	2

Abbreviations: AUROC = area under receiver operating characteristic curve; CI = confidence interval; SVM = support vector machine; LR = logistic regression; RF = random forest.

## Data Availability

The raw data used in this study remains confidential and will not be shared due to China Medical University Hospital policy.

## Supplementary Materials

The following supporting information can be downloaded at: https://www.mdpi.com/article/10.3390/diagnostics15212790/s1, Table S1: List of inputted features. Table S2: ICD-9-CM and ICD-10-CM codes used to identify cirrhosis and complications in HBV-related cirrhosis patients. Table S3: Performance metrics of the machine learning models. Table S4: Characteristic profile of balanced patient samples used in predicting variceal bleeding. Table S5: Characteristic profile of balanced patient samples used in predicting ascites. Table S6: Characteristic profile of balanced patient samples used in predicting jaundice. Table S7: Characteristic profile of balanced patient samples used in predicting multiple complications. Table S8: Selected features for each model.

## Abbreviations

The following abbreviations are used in this manuscript:

AFP	alpha-fetoprotein
ALT	alanine aminotransferase
ANN	Artificial Neural Network
Anti-HBe	anti-hepatitis B e-antigen
Anti-HBs	anti-hepatitis B surface antibody
AST	aspartate transferase
AUROC	area under the receiver operating characteristic curve
CHB	chronic hepatitis B
CI	confidence interval
CNN	Convolutional Neural Network
DNN	Deep Neural Network
DT	Decision Tree
ETV	entecavir
HBeAg	hepatitis B e antigen
HBsAg	hepatitis B surface antigen
HBV	hepatitis B virus
HCC	hepatocellular carcinoma
HCV	hepatitis C virus
HE	hepatic encephalopathy
HRS	hepatorenal syndrome
ICD-9	International Classification of Diseases, Ninth Revision Clinical Modification
ICD-10	International Classification of Diseases, Tenth Revision Clinical Modification
LAM	lamivudine
LASSO	Least Absolute Shrinkage and Selection Operator
LR	Logistic Regression
ML	machine learning
PT	prothrombin time
RF	Random Forest
SBP	spontaneous bacterial peritonitis
SVM	Support Vector Machine
TDF	tenofovir
XGBoost	Extreme Gradient Boosting
