# Supplementary material for: Machine Learning-Based Prediction of Decompensation in Hepatitis B Virus-Related Cirrhosis
Source: Diagnostics (Basel). 2025 Nov 4;15(21):2790. doi: 10.3390/diagnostics15212790 (PMC12610556; doi:10.3390/diagnostics15212790)
Supplement: Supplementary file 1 [file diagnostics-15-02790-s001.zip › diagnostics-3937561-supplementary.pdf]

Table S1. List of inputted features.

	Type	Feature name
1	Patient demographic	Sex
2	Patient demographic	Age
3	Liver biochemical test	Alanine aminotransferase; ALT[1]
4	Liver biochemical test	Aspartate aminotransferase; AST[1]
5	Liver biochemical test	Total bilirubin[2]
6	Liver biochemical test	Albumin[3]
7	Liver biochemical test	Prothrombin time; PT[4]
8	Liver biochemical test	Platelet count[5]
9	Other related laboratory test	Creatinine[6]
10	Other related laboratory test	Sodium[7]
11	Other related laboratory test	Fasting glucose[8]
12	Other related laboratory test	Alpha-fetoprotein; AFP[9]
13	Medication adherence	Length of treatment (in days)[10]
14	Medication adherence	Medication possession ratio; MPR

[1] Alanine aminotransferase (ALT) and aspartate aminotransferase (AST) - These liver enzymes are sensitive indicators of liver cell injury. Elevated levels usually indicate liver cell or tissue damage and are common in most liver diseases.

[2] Total bilirubin – A byproduct of hemoglobin breakdown, bilirubin is processed by the liver and excreted in bile. Elevated bilirubin levels indicate liver dysfunction and can cause jaundice. Higher serum bilirubin in viral hepatitis suggests greater liver damage and a longer disease course. High levels of bilirubin can also cause jaundice.

[3] Albumin – A liver-produced protein that prevents fluid leakage from blood vessels. Low albumin levels can cause fluid accumulation in the abdomen (ascites) or legs (edema).

[4] Prothrombin time (PT) - Measures how long blood takes to clot. A high PT indicates delayed clotting due to insufficient clotting proteins from the liver, suggesting serious liver damage or cirrhosis and a higher risk of internal bleeding. Serum concentrations of bilirubin and albumin, and prothrombin time are key parameters in the Child-Turcotte-Pugh score which can be used to estimate liver disease severity.

[5] Platelet count - Platelets help blood clot. Cirrhosis patients often have low platelet counts, leading to easier bruising or bleeding.

[6] Creatinine – A marker for kidney function, creatinine is produced from the breakdown of muscle protein. High levels indicate impaired kidney function, which can suggest renal insufficiency, a common complication in cirrhosis patients (e.g., hepatorenal syndrome).

[7] Sodium (Na) – A biomarker for detecting hyponatremia in cirrhosis patients with ascites; a key component in calculating the Model for End-stage Liver Disease-Sodium (MELD-Na) score, which assesses liver cirrhosis severity.

[8] Fasting glucose – Elevated fasting glucose levels in chronic liver disease patients can indicate impaired glucose tolerance and fluctuating glucose levels, signifying deteriorating liver function. Impaired liver function can also impede glucose release from glycogen storage, causing hypoglycemia, an important prognostic factor for cirrhotic patients.

[9] Alpha-fetoprotein (AFP) - A protein found in liver disease patients, AFP serves as a tumor marker. High levels may indicate liver cancer.

[10] The length of treatment and treatment compliance, calculated using the medication possession ratio, reflect how well patients follow doctors' orders. Poor compliance generally leads to weaker treatment effectiveness and poor disease prognosis.

Note: Medication possession ratio (MPR) is calculated as a ratio of the number of days the patient has supply of medication over the total number of observed days. A MPR of greater than 0.8 is indicative of highly adherent behavior and a MPR of 0 indicates no use.

Table S2. ICD-9-CM and ICD-10-CM codes used to identify cirrhosis and complications in HBV-related cirrhosis patients.

Condition	ICD-9-CM codes	ICD-10-CM codes
Cirrhosis	571.5	K74.0、K74.60、K74.69
Ascites	568.82、789.5	R18.8
Variceal bleeding	456.2、456.2、578、578.1、578.9	I85.01、I85.11、K290、K291、K922
Jaundice	782.4	R17
Multiple complications	Diagnosed with two or more complications concurrently; complications may include ascites, variceal bleeding, jaundice, hepatic encephalopathy, hepatorenal syndrome, or spontaneous bacterial peritonitis.	

Table S3. Performance metrics of the machine learning models.

Complication	Metric	LAM Medication				ETV Medication			
		SVM	LR	DT	RF	SVM	LR	DT	RF
Variceal bleeding	CV_accuracy	0.68	0.61	0.59	0.65	0.69	0.66	0.66	0.60
	Accuracy	0.58	0.64	0.57	0.61	0.70	0.65	0.71	0.64
	AUC	0.65	0.63	0.59	0.63	0.71	0.61	0.64	0.61
	Sensitivity	0.76	0.61	0.62	0.66	0.71	0.56	0.54	0.56
	Specificity	0.55	0.65	0.56	0.60	0.70	0.67	0.73	0.65
	F1-score	0.40	0.39	0.35	0.39	0.38	0.29	0.32	0.28
	MCC	0.23	0.20	0.14	0.20	0.29	0.16	0.14	0.20
Ascites	CV_accuracy	0.70	0.62	0.48	0.61	0.74	0.73	0.70	0.77
	Accuracy	0.60	0.70	0.60	0.66	0.63	0.72	0.64	0.70
	AUC	0.69	0.69	0.55	0.69	0.74	0.73	0.71	0.77
	Sensitivity	0.77	0.68	0.50	0.73	0.86	0.75	0.79	0.86
	Specificity	0.60	0.70	0.60	0.65	0.62	0.72	0.64	0.69
	F1-score	0.12	0.14	0.08	0.13	0.12	0.14	0.12	0.15
	MCC	0.14	0.15	0.04	0.15	0.17	0.17	0.15	0.20
Jaundice	CV_accuracy	0.83	0.78	0.73	0.73	0.83	0.74	0.81	0.85
	Accuracy	0.68	0.69	0.63	0.67	0.76	0.76	0.73	0.69
	AUC	0.74	0.71	0.68	0.77	0.70	0.63	0.73	0.71
	Sensitivity	0.80	0.73	0.73	0.87	0.64	0.50	0.73	0.73
	Specificity	0.67	0.69	0.63	0.66	0.76	0.77	0.72	0.69
	F1-score	0.10	0.10	0.09	0.11	0.11	0.09	0.11	0.10
	MCC	0.15	0.13	0.11	0.17	0.14	0.09	0.15	0.13
Multiple complications	CV_accuracy	0.74	0.74	0.67	0.76	0.76	0.75	0.72	0.78
	Accuracy	0.63	0.68	0.65	0.66	0.78	0.75	0.71	0.74
	AUC	0.69	0.72	0.70	0.71	0.75	0.73	0.68	0.77
	Sensitivity	0.75	0.75	0.75	0.78	0.71	0.71	0.65	0.80
	Specificity	0.62	0.68	0.64	0.65	0.78	0.75	0.71	0.74
	F1-score	0.22	0.25	0.23	0.24	0.27	0.25	0.21	0.27
	MCC	0.19	0.23	0.21	0.22	0.27	0.24	0.18	0.28

Note: CV_accuracy = the best accuracy in 10-fold cross validation.

Abbreviations: LAM – lamivudine; ETV – entecavir; AUC = area under curve.

Table S4. Characteristic profile of balanced patient samples used in predicting variceal bleeding.

	LAM				ETV			
	Compensated (n=326)		Decompensated (n=326)		Compensated (n=374)		Decompensated (n=374)	
	Mean (n)	SD (%)	Mean (n)	SD (%)	Mean (n)	SD (%)	Mean (n)	SD (%)
Sex								
Male	**239**	73.31	**248**	76.07	**266**	71.12	**289**	77.27
Female	**87**	26.69	**78**	23.93	**108**	28.88	**85**	22.73
Age (year)	**51.77**	12.11	**51.94**	12.94	**51.1**	11.92	**52.15**	13.07
ALT	**37.68**	18.06	**37.65**	18.03	**37.38**	18.53	**39.81**	19.78
AST	**49.95**	27.05	**60.37**	30.04	**46.37**	24.82	**56.87**	27.05
Total bilirubin	**1.29**	0.72	**1.49**	0.82	**1.22**	0.57	**1.40**	0.74
Albumin	**3.51**	0.88	**3.12**	0.79	**3.65**	0.88	**3.39**	0.84
Fasting glucose	**125.82**	36.63	**132.93**	36.48	**130.36**	40.02	**135.81**	38.63
Creatinine	**0.95**	0.26	**0.97**	0.29	**0.92**	0.26	**0.94**	0.27
Prothrombin time	**13.38**	2.84	**14.26**	2.55	**12.65**	2.19	**13.78**	2.43
Sodium	**136.61**	3.37	**136.35**	3.66	**136.71**	3.14	**136.88**	3.49
Platelet count	**126.37**	60.44	**107.05**	59.49	**128.07**	58.62	**108.65**	57.94
AFP	**4.33**	3.19	**4.47**	3.14	**4.20**	2.68	**4.72**	3.11
Length of treatment (day)	**69.01**	366.6	**36.39**	170.3	**355.9**	835.8	**84.27**	277.8
MPR								
None	**284**	87.12	**284**	87.12	**273**	72.99	**273**	72.99
Low	**42**	12.88	**9**	2.76	**101**	27.01	**28**	7.49
High	**0**	0	**33**	10.12	**0**	0	**73**	19.52

Abbreviations: LAM – lamivudine; ETV – entecavir; SD – standard deviation; ALT - alanine aminotransferase; AST - aspartate aminotransferase; AFP - alpha-fetoprotein; MPR - medication possession ratio.

Table S5. Characteristic profile of balanced patient samples used in predicting ascites.

	LAM					ETV			
	Compensated (n=89)		Decompensated (n=89)			Compensated (n=111)		Decompensated (n=111)	
	Mean (n)	SD (%)	Mean (n)	SD (%)		Mean (n)	SD (%)	Mean (n)	SD (%)
Sex									
Male	**64**	71.91	**57**	64.04		**85**	76.58	**72**	64.86
Female	**25**	28.09	**32**	35.96		**26**	23.42	**39**	35.14
Age (year)	**52.38**	12.47	**55.12**	12.85		**51.14**	11.58	**56.04**	12.41
ALT	**33.70**	16.72	**41.30**	19.95		**35.11**	16.68	**44.57**	21.12
AST	**45.15**	24.98	**59.16**	30.02		**43.12**	23.60	**57.40**	25.98
Total bilirubin	**1.22**	0.68	**1.54**	0.78		**1.25**	0.67	**1.43**	0.72
Albumin	**3.73**	0.84	**2.90**	0.71		**3.80**	0.79	**3.00**	0.68
Fasting glucose	**127.93**	36.71	**127.97**	39.52		**131.77**	40.48	**129.50**	41.95
Creatinine	**0.94**	0.24	**1.01**	0.34		**0.94**	0.27	**0.95**	0.28
Prothrombin time	**12.94**	2.68	**14.26**	2.58		**12.26**	1.71	**13.72**	2.48
Sodium	**136.95**	3.08	**135.46**	3.86		**137.02**	3.15	**134.77**	3.57
Platelet count	**131.7**	63.2	**110.29**	51.64		**131.82**	55.76	**110.88**	55.30
AFP	**4.48**	3.07	**5.36**	3.80		**4.02**	2.49	**5.18**	3.66
Length of treatment (day)	**46.04**	260.3	**5.96**	27.73		**417.36**	871.8	**31.30**	137.0
MPR									
None	**80**	89.89	**80**	89.89		**74**	66.67	**74**	66.67
Low	**9**	10.11	**1**	1.12		**37**	33.33	**14**	12.61
High	**0**	0	**8**	8.99		**0**	0	**23**	20.72

Abbreviations: LAM – lamivudine; ETV – entecavir; SD – standard deviation; ALT - alanine aminotransferase; AST - aspartate aminotransferase; AFP - alpha-fetoprotein; MPR - medication possession ratio.

Table S6. Characteristic profile of balanced patient samples used in predicting jaundice.

	LAM					ETV			
	Compensated (n=60)		Decompensated (n=60)			Compensated (n=86)		Decompensated (n=86)	
	Mean (n)	SD (%)	Mean (n)	SD (%)		Mean (n)	SD (%)	Mean (n)	SD (%)
Sex									
Male	**43**	71.67	**48**	80.00		**63**	73.26	**68**	79.07
Female	**17**	28.33	**12**	20.00		**23**	26.74	**18**	20.93
Age (year)	**50.88**	11.00	**53.25**	13.86		**51.58**	12.03	**52.36**	12.49
ALT	**39.67**	18.07	**48.43**	22.40		**41.51**	21.84	**50.00**	25.98
AST	**53.41**	30.51	**70.46**	33.10		**42.97**	19.46	**60.36**	30.71
Total bilirubin	**1.33**	0.92	**2.14**	1.21		**1.21**	0.63	**1.75**	0.94
Albumin	**3.51**	1.00	**3.07**	0.72		**3.84**	0.86	**3.35**	0.72
Fasting glucose	**122.03**	37.94	**137.5**	41.17		**134.31**	38.36	**140.20**	46.28
Creatinine	**0.96**	0.29	**0.93**	0.30		**0.90**	0.22	**0.93**	0.29
Prothrombin time	**12.84**	2.59	**14.64**	3.11		**11.80**	1.46	**13.36**	2.31
Sodium	**137.17**	2.96	**134.93**	3.81		**137.44**	3.28	**135.48**	3.07
Platelet count	**133.06**	53.08	**122.16**	62.94		**137.81**	52.58	**127.42**	62.03
AFP	**4.55**	3.44	**4.03**	2.81		**3.99**	2.86	**4.12**	2.56
Length of treatment (day)	**150.28**	641.7	**28.4**	104.7		**573.52**	871.9	**96.49**	354.2
MPR									
None	**47**	78.33	**47**	78.33		**40**	46.51	**40**	46.51
Low	**13**	21.67	**1**	1.67		**46**	53.49	**7**	8.14
High	**0**	0	**12**	20.00		**0**	0	**39**	45.35

Abbreviations: LAM – lamivudine; ETV – entecavir; SD – standard deviation; ALT - alanine aminotransferase; AST - aspartate aminotransferase; AFP - alpha-fetoprotein; MPR - medication possession ratio.

Table S7. Characteristic profile of balanced patient samples used in predicting multiple complications.

	LAM				ETV			
	Compensated (n=161)		Decompensated (n=161)		Compensated (n=202)		Decompensated (n=202)	
	Mean (n)	SD (%)	Mean (n)	SD (%)	Mean (n)	SD (%)	Mean (n)	SD (%)
Sex								
Male	**112**	69.57	**136**	84.47	**144**	71.29	**168**	83.17
Female	**49**	30.43	**25**	15.53	**58**	28.71	**34**	16.83
Age (year)	**51.57**	12.07	**55.84**	12.94	**51.56**	11.75	**55.25**	12.97
ALT	**40.51**	22.37	**45.14**	24.92	**39.53**	19.29	**47.07**	24.60
AST	**49.10**	27.90	**63.96**	34.36	**46.93**	24.91	**63.05**	32.06
Total bilirubin	**1.39**	0.94	**1.91**	1.17	**1.25**	0.68	**1.73**	1.01
Albumin	**3.54**	0.85	**2.93**	0.77	**3.79**	0.82	**3.02**	0.82
Fasting glucose	**125.73**	40.02	**131.10**	36.96	**129.84**	41.19	**137.40**	41.53
Creatinine	**0.97**	0.34	**0.99**	0.34	**0.90**	0.23	**0.98**	0.32
Prothrombin time	**13.21**	2.98	**15.66**	3.54	**12.44**	2.21	**14.43**	2.83
Sodium	**136.03**	3.80	**134.30**	4.39	**136.48**	3.35	**134.89**	4.31
Platelet count	**122.60**	57.62	**113.26**	56.86	**128.35**	50.99	**111.69**	61.29
AFP	**4.78**	3.84	**5.42**	3.78	**4.23**	2.97	**4.45**	3.00
Length of treatment (day)	**41.98**	256.7	**1.42**	5.65	**424.78**	847.3	**9.21**	23.30
MPR								
None	**150**	93.17	**150**	93.17	**139**	68.81	**139**	68.81
Low	**11**	6.83	**2**	1.24	**63**	31.19	**16**	7.92
High	**0**	0	**9**	5.59	**0**	0	**47**	23.27

Abbreviations: LAM – lamivudine; ETV – entecavir; SD – standard deviation; ALT - alanine aminotransferase; AST - aspartate aminotransferase; AFP - alpha-fetoprotein; MPR - medication possession ratio.

Table S8. Selected features for each model.

Complication	LAM	ETV
Variceal bleeding	AST, total bilirubin, albumin, fasting glucose, creatinine, prothrombin time, platelet count, MPR (8)	Sex, AST, total bilirubin, albumin, fasting glucose, creatinine, prothrombin time, platelet count, AFP, length of treatment, MPR (11)
Ascites	Age, AST, total bilirubin, albumin, creatinine, prothrombin time, sodium, platelet count, AFP, MPR (10)	Age, ALT, AST, total bilirubin, albumin, prothrombin time, sodium, platelet count, AFP, length of treatment (10)
Jaundice	ALT, AST, total bilirubin, albumin, fasting glucose, prothrombin time, sodium, length of treatment, MPR (9)	ALT, AST, total bilirubin, albumin, fasting glucose, prothrombin time, sodium, length of treatment, MPR (9)
Multiple complications	Sex, age, ALT, AST, total bilirubin, albumin, fasting glucose, prothrombin time, sodium, platelet count, length of treatment, MPR (12)	Sex, age, ALT, AST, total bilirubin, albumin, fasting glucose, creatinine, prothrombin time, sodium, platelet count, length of treatment, MPR (13)

Note: The most frequently selected features include AST, total bilirubin, albumin, and prothrombin time, present in all complications for both medication groups. MPR is the second most selected feature, only ablated in predicting ascites among ETV users.

Abbreviations: LAM = lamivudine; ETV = entecavir; HCC = hepatocellular carcinoma; AST = aspartate aminotransferase; ALT = alanine aminotransferase; AFP = alpha-fetoprotein; MPR = medication possession ratio.
